# Differential Expression of Circadian Genes in Leukemia and a Possible Role for Sirt1 in Restoring the Circadian Clock in Chronic Myeloid Leukemia

**DOI:** 10.5334/jcr.147

**Published:** 2017-04-28

**Authors:** Sabhi Rahman, Al-Shaimaa Al-hallaj, Atef Nedhi, Gmal Gmati, khadega Ahmed, Haya Al Jama, Thadeo Trivilegio, Abdullah Mashour, Ahmad Al Askar, Mohamed Boudjelal

**Affiliations:** 1 Drug Discovery Unit-Core Facility Medical Platforms and Technology, King Abdullah International Medical Research Centre (KAIMRC), National Guard Health Affairs, Riyadh, SA; 2 King Saud Bin Abdulaziz University for health Sciences, Riyadh, SA; 3 Ministry of National Guard Health Affairs, Riyadh, SA

**Keywords:** Circadian Clock, Leukemia, Sirt1, Reverb a, Bmal, Per2

## Abstract

Disregulation of genes making up the mammalian circadian clock has been associated with different forms of cancer. This study aimed to address how the circadian clock genes behave over the course of treatment for both the acute and chronic forms of leukemia and whether any could be used as potential biomarkers as a read-out for therapeutic efficacy. Expression profiling for both core and ancillary clock genes revealed that the majority of clock genes are down-regulated in acute myeloid leukemia patients, except for Cry2, which is up-regulated towards the end of treatment. A similar process was seen in acute lymphocytic leukemia patients; however, here, Cry2 expression came back up towards control levels upon treatment completion. In addition, all of the core clock genes were down-regulated in both chronic forms of leukemia (chronic myeloid leukemia and chronic lymphocytic leukemia), except for Cry2, which was not affected when the disease was diagnosed. Furthermore, the NAD(+) – dependent protein deacetylase Sirt1 has been proposed to have a dual role in both control of circadian clock circuitry and promotion of cell survival by inhibiting apoptotic pathways in cancer. We used a pharmacological-based approach to see whether Sirt1 played a role in regulating the circadian clock circuitry in both acute and chronic forms of leukemia. Our results suggest that interfering with Sirt1 leads to a partial restoration of BMAL1 oscillation in chronic myeloid leukemia patient samples. Furthermore, interfering with Sirt1 activity led to both the induction and repression of circadian clock genes in both acute and chronic forms of leukemia, which makes it a potential therapeutic target to either augment existing therapies for chronic leukemia or to act as a means of facilitating chronotherapy in order to maximize both the effectiveness of existing therapies and to minimize therapy-associated toxicity.

## Introduction

Important mammalian physiological processes such as sleep-wake patterns, gene transcription and regulation of metabolism to name a few; undergo periodic changes or circadian rhythms which typically span a 24h cycle [1,2,3]. Two components underpin the circadian system in mammals: A central pacemaker resident within the suprachiasmatic nucleus (SCN) found in the anterior hypothalamus together with core circadian genes that collectively make up the circadian oscillator and secondly peripheral oscillators located in different tissues around the body [3,4]. In order for an organism to respond appropriately to light and dark cycles, a fully functional circadian clock is of paramount importance. Circadian oscillators make use of transcriptional-translational feedback loops that rely on both positive and negative cues from oscillators which in turn causes the characteristic daily cycling of both clock genes as well as clock controlled genes [5] (Yang and Sheng-Fun, 2016). In addition, epigenetics also plays a critical role in regulating the daily periodicity of the circadian clock [6].

Disruption in circadian rhythms appears to be a hallmark for a number of diseases including diabetes and cancer [7]. There is an accumulating body of evidence showing a link between disruption of the circadian clock and pathogenesis of cancer. *Per1* and *Per2* are down-regulated in both sporadic and familial forms of breast cancer when compared to normal breast tissue [8]. In leukemia, a progressive, malignant disorder that affects the bone marrow and other blood-forming organs resulting in the abnormal production of leukocytes, changes in circadian genes have also been reported. For example, epigenetic changes through the hyper-methylation of the *BMAL1* promoter has been associated with the development of hematological malignancies such as non-Hodgkin lymphoma and acute lymphocytic leukemia (ALL), and hyper-methylation of the *Per3* gene is manifested in chronic myeloid leukemia (CML) in peripheral blood [9]. A comprehensive understanding of how circadian genes behave in leukemia will not only provide insights into how the molecular clock is impacted in this disease but such an understanding could also be used to develop treatment strategies whereby drugs are administered to patients in leukemia in synchrony with their molecular clocks to facilitate greater effectiveness of drug administration to the patients (a process known as chronotherapy).

Although altered expression of circadian genes has been previously reported in chronic and acute leukemia [9,10,11], no study has looked at how circadian genes change over the course of treatment for leukemia and during disease relapse in the case of AML and upon diagnosis and during the course of treatment for chronic lymphocytic leukemia (CLL) and whether these genes could be used as biomarkers to assess therapeutic efficacy. Furthermore, we currently lack an understanding of how the oscillation of clock genes is altered in both acute and chronic forms of leukemia and what role if any sirtuin (*Sirt1*) has in regulating the circadian clock in leukemia given that *Sirt1*, an NAD(+) – dependent protein deacetylase, has previously been shown to regulate circadian clock gene expression in other cellular systems by directly binding to CLOCK-BMAL1, which in turn promotes both the de-acetylation and degradation of *Per2* [12].

In the present study we first investigated the changes in circadian clock gene expression across different forms of leukemia at the point of disease diagnosis, upon treatment completion and in the case of AML during disease relapse with a view to identifying circadian genes as potential biomarkers that could be used to assess treatment efficacy. Secondly, the oscillation of core and ancillary circadian genes were analyzed in both acute and chronic forms of leukemia. Finally, we probed the role of *Sirt1* in regulating circadian gene expression *in vitro* using a small molecule approach in primary cells derived from patients representing both acute and chronic forms of leukemia to see whether *Sirt1* it has any role in regulating the circadian clock in both acute and chronic forms leukemia.

## Materials and Methods

### Patients, healthy individuals and samples

Peripheral blood samples were collected from patients and healthy individuals at the Ministry of National Guard Health Affairs (MNGHA), King Abdulaziz Medical City, Riyadh following signed consent after IRB approval and according to guidelines established at MNGHA. In total the following numbers of patients were recruited for the study, which was spread across different stages of the disease and treatment: 26 patients with acute myeloid leukemia (AML), 22 patients with acute lymphoid leukemia (ALL), 13 patients with chronic myeloid leukemia (CML) and 14 patients with chronic lymphoid leukemia (CLL). In addition, 30 healthy donors (controls) were also recruited for comparative data analysis purposes. Peripheral blood mononuclear cells (PBMCs) were isolated from peripheral blood using Leucosep tubes (Greiner Bio-One) according to a previously described method [13]. The blood withdrawal from patients and PBMCs preparation were performed between 8 and 10 o’clock in the morning, as all the patients came to the clinic in the morning and also for us to avoid a time shift issue between samples.

### Cell culture and compound treatment

PBMCs used in this study were maintained and propagated in DMEM media (UCF) supplemented with 2 mM glutamine, 1X non-essential amino acids, 100 U/ml penicillin, 100 U/ml streptomycin and 10% fetal bovine serum (ATCC). All cells were incubated in a humidified atmosphere of 5% CO_2_ and 95% air at a constant temperature of 37°C unless otherwise specified. The *Sirt1* inhibitor used in this study, EX527, was purchased from TOCRIS and was received in lyophilized form. 100 mM stocks of the inhibitor were prepared using dimethyl sulphoxide (DMSO). Working concentration of EX527 (30 mM) was added to cells directly in culture media. All compound treatments were performed in 6, 12 or 24-well plates (Corning).

### RNA isolation and synthesis of cDNA

Total RNA from each sample was extracted using the PureLink RNA mini kit (ThermoFisher Scientific) according to the manufacturer’s instructions. Following extraction, the quantity and quality was assessed using the Nanodrop 8000 8-well spectrophotometer (ThermoFisher). First-strand synthesis was performed on 400 ng of total RNA using the high capacity cDNA Reverse Transcription Kit (Applied Biosystems) according to the manufacturers recommendation using the following PCR cycling parameters: 25°C for 10mins, 37°C for 120mins, 85°C for 5mins and finally held at 4°C.

### Quantitative reverse-transcription-polymerase chain reaction (qRT-PCR) analysis of mammalian circadian genes

The expression of seven circadian clock genes *Per2, BMAL1, Cry1, Cry2, Clock, REV-ERBa* and *PPARa* as well as two circadian-modifier genes, *Sirt1* and *c-myc*, were analyzed using Taqman expression assays (Table 1). Glyceraldehye-3-phosphate dehydrogenase (GAPDH) expression was used as the internal control. All reactions were performed in a MicroAmp optical 384-well plate (Applied Biosystems) in a final volume of 5 ul per reaction. For each reaction, 1 ul of CDNA was used as the input for amplification.

**Table 1:** Taqman assays used for qRT-PCR gene expression analysis.

Gene	Taqman Assay	Interrogated sequence (RefSeq)	Amplicon length

Per2	Hs00256143_m1	NM_022817.2	121
BMAL1/ARNTL	Hs00154147_m1	NM_001030272.2	112
CRY1	Hs00172734_m1	NM_004075.4	84
CRY2	Hs00323654_m1	NM_001127457.2	75
CLOCK	Hs00231857_m1	NM_001267843.1	88
REV-ERBa/NR1D1	Hs00253876_m1	NM_021724.4	60
PPARa	Hs00947536_m1	NM_001001928.2	62
SIRT1	Hs01009006_m1	NM_001142498.1	91
c-myc	Hs00153408_m1	NM_002467.4	107

Amplification was performed using the 7900 HT Fast Real-time PCR system (Thermofisher) and the PCR cycling parameters were as follows: 95°C for 10mins followed by 40 cycles of PCR reactions at 95°C for 30 sec and 60°C for 1 min. The relative expression levels of the genes assayed in the cell synchronization validation method experiment were calculated using the comparative threshold cycle Ct (ΔΔCt) method. Initially, the Ct value of each gene was normalized to the corresponding Ct value of GAPDH for the same sample to obtain the relative threshold cycle (ΔCt). Following this the ΔCt for each biological replicate was then exponentially transformed into ΔCt expression by calculating 2 raised to the -ΔCt. Next, the average and standard deviation of the biological replicates were calculated followed by normalization to the average of the control samples for the same gene (ΔΔCt). Finally the ΔΔCt was calibrated from control samples and expressed as a relative fold-change. For relative mRNA analysis, raw Ct values were exported from ABI 7900 and imported into Microsoft Excel. Ct values were then used to calculate copy number for each well using historical/generic values from standard curves (Intersection at 0 = 40, slope = –3.5) (Data not shown). Copy numbers were then normalized to GAPDH and mRNA levels were expressed relative to control samples.

### Cell synchronization

PBMC populations were synchronized using three different methods. One involved culturing cells initially in media containing 10% Fetal Bovine Serum (FBS), another involved culturing cells in 50% FBS for 1hr at 37°C/5% CO_2_ followed by culture in 10% FBS, and the third method involved culturing the cells at 32°C for 12 hours followed by 37°C culture for 12 additional hours before use.

### Statistical analysis

Statistical differences between clock gene expression in leukemia patients and control samples were determined using the student’s *t-test* and assuming unequal variances. P-values < *0.05, **0.01 and ***0.001 were considered to be statistically significant. Expression in patient samples was expressed relative to control samples (control samples were given an arbitrary value of 1 and patient samples were calibrated to this).

## Results

### The temperature change method was more effective at synchronizing cell populations

Different methods exist for the synchronization of cells within a given population. These include culturing cells under serum-starved conditions to enrich for cells in the G_0_ phase of the cell cycle [14,15] and lowering the temperature of cell culture to 30–32°C to arrest cells in the G_1_ phase of the cell cycle [16]. Other approaches for cell synchronization make use of pharmacological agents that induce cell cycle arrest. For example, treatment with mimosine prevents DNA replication by interfering with replication origins and induces G_1_ arrest [17,18] or treatment with hydroxyurea induces S-page arrest by inhibition of an enzyme involved in the biosynthesis of deoxyribonucleotides [19]. We decided against taking a pharmacological-based approach for cell synchronization given the possibility of this perturbing clock gene expression.

For our study we decided to evaluate three different methods for synchronizing PBMC populations. One involved culturing cells in 10% FBS, another involved culturing cells in 50% FBS followed by switching to 10% FBS and the third involved culturing cells at a lower culture temperature of 32°C for 12 hours followed by a return to 37°C before use. Reducing the temperature of cultures has previously been shown in fibroblasts to slow down cell cycle kinetics and thus enables cells to remain within the G_0_ phase of the cell cycle [16].

**Figure 1:**
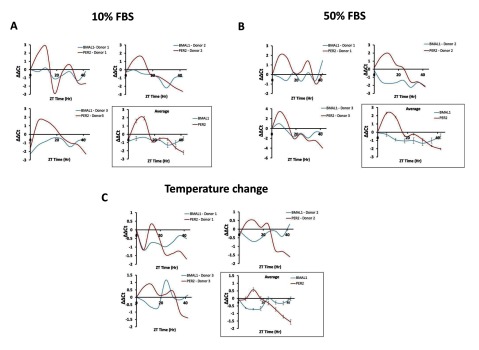
**Temperature change is a more suitable method for synchronization of human PBMC populations than using 10% or 50% FBS in cell culture.** PBMC’s were synchronized either by culturing in 10% FBS for 12 hours followed by sample collection **(A)** or by culturing in 50% FBS **(B)** or by culturing cells at the reduced temperature of 32°C for 12 hours followed by 12 hours of culture at 37°C for a further 12 hours before sample collection **(C)**. In each experiment, RNA samples were harvested from cell pellets from three different donors at 6hr intervals over a period of 42hrs for qRT-PCR analysis *BMAL1* and *Per2* expression over this period. ΔΔCt analysis was performed in SDS 5.4 (Applied Biosystems). GAPDH was used as a reference control for all analyses. Average expression across the three donor samples for each synchronization method tested is shown in boxes.

As Figure 1A shows in the average expressing across three different donors, culturing the cells continuously in 10% FBS failed to show any evidence of oscillation of *Per2* and *BMAL1* suggesting that the cell population were probably in an asynchronous state. Culturing the cells in high FBS (50%) followed by culture in 10% FBS, appeared to show some evidence of oscillation (Figure 1B) which was consistent with what has been previously described in human PBMC samples [20]. However, the large variability seen in these samples suggested that the synchronization of the cells was sub-optimal at best. We found that the temperature change method gave oscillation patterns consistent with what has been previously reported. Per2 expression peaked between 6 and 12 hours whereas BMAL1 expression showed a maximum in the opposite direction during the same time period. Furthermore, at 24 hours, the oscillation patterns of the two circadian genes switched over. Based on these findings all subsequent experiments were performed on cell samples following synchronization using the temperature change method.

### Circadian genes show distinct patterns of expression during diagnosis of AML, upon treatment completion, and Cry2 is up-regulated during disease relapse

**Figure 2:**
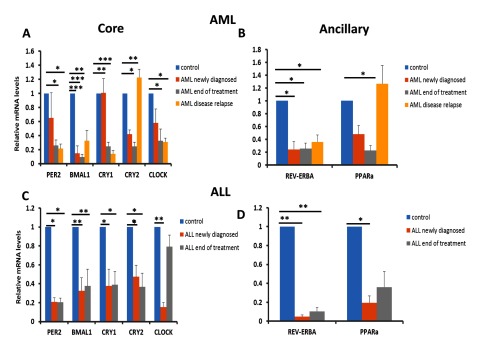
**Core and ancillary circadian genes are down-regulated in PBMCs derived from patients diagnosed with either AML or ALL and show further changes in expression at the end of treatment and during disease relapse.** RNA samples from PBMC cells were harvested directly from patients newly diagnosed with AML or ALL, towards the end of treatment for both AML or ALL and during disease relapse for AML patients. The core clock genes including *Per2, BMAL1, Cry1, Cry2 and clock*; and the ancillary clock genes *Rev-ERBa and PPARa* were examined by qRT-PCR. Arbitrary copy numbers were calculated, normalized to GAPDH and calibrated relative to control samples (control samples were given a value of 1). Mean mRNA levels are plotted relative to control samples (mean ± SD). Statistical differences were determined by student’s t-test assuming unequal variances. Statistical significance at *p < 0.05, **p < 0.01 and ***p < 0.001.

PBMC samples were collected from a total of 26 patients spread out across different stages of the disease (9 samples were derived from patients that had been newly diagnosed with AML, 8 were from patients that had completed treatment and 9 were from patients in whom the disease had relapsed. From these samples total RNA was isolated, reverse-transcribed in to cDNA and amplified by quantitative PCR using probes designed to 7 circadian genes (*Per2, BMAL1, Cry1, Cry2, Clock, REV-ERBa* (Table 1). Our data demonstrated that upon diagnosis of AML, the core circadian clock genes are down regulated with the exception of CRY1 which is not affected in newly diagnosed patients. Bmal1 is the most affected gene followed by Cry2, Clock and then the Per2 gene. In addition the ancillary clock gene, Rev-ERBa (p < 0.05) is significantly down-regulated. PPARa is also down regulated in new and end of treatment AML when compared to samples from healthy individuals (Figure 2B). This is in concordance with a previous study that has shown significant down-regulation in core clock genes in newly-diagnosed AML patients [11]. Upon completion of treatment, the core clock genes Cry1 (p < 0.01), Cry2 (p < 0.05) and CLOCK (p < 0.05) showed significant down-regulation compared to controls. The same was also observed for the two ancillary clock genes tested; *REV-ERBa* (p < 0.05) and *PPARa* (p < 0.05). Furthermore, upon relapse of the disease, some of the core clock genes showed significant down-regulation; *Per2* (p < 0.05), *BMAL1* (p < 0.05), Cry1 (p < 0.001), and *Clock* (p < 0.05) as well as the ancillary clock gene, *REV-ERBa* (p < 0.01). Interestingly, Cry2 (p < 0.01) showed significant up-regulation in disease relapse patients compared to control samples. The significant down-regulation of core and ancillary clock genes seen in AML patients at the end of treatment gives rise to the possibility of these genes being used as potential biomarkers to assess efficacy in the treatment of AML and the up-regulation of Cry2 could be used as a potential biomarker to assess relapse status in AML before other diagnostic tests are carried out to confirm that the disease has relapsed in patients.

### Expression of the CLOCK gene is restored to control levels in ALL patients after treatment

We next wanted to see whether the observations in circadian gene expression seen in AML patients were also reflected in patients diagnosed with ALL to determine whether similarities existed between the acute forms of both myeloid and lymphoid leukemia. For this reason, 22 patient samples (13 patients newly diagnosed with ALL and 9 patients at the end of treatment) were expression profiled by qRT-PCR for the circadian clock genes. Here we observed complete down regulation of all the core clock genes tested: Per2 (p < 0.05), BMAL1 (p < 0.01), Cry1 (p < 0.05), Cry2 (P < 0.05) and CLOCK (p < 0.01) (Figure 2C) as well as the two ancillary clock genes tested: Rev-ERBa (p < 0.01) and PPARa (p < 0.05) (Figure 2D). Upon completion of treatment none of the core clock genes gave significant differences in expression when compared to the expression in newly diagnosed ALL patients. However, significant differences at the completion of treatment were seen when compared to control patient samples: Per2 (p < 0.05), BMAL1 (p < 0.01), Cry1 (p < 0.05) and Cry2 (p < 0.01). A similar observation was also observed for Rev-ERBa (p < 0.01). Interestingly, the only core clock gene that showed significant changes in expression in patient samples following treatment completion was CLOCK. Levels of CLOCK were observed to have returned to levels seen in healthy patients (Figure 2C). This observation with CLOCK at the end of treatment raises two possibilities. One possibility is that CLOCK expression in ALL patients is more sensitive to therapy or the other possibility is that CLOCK expression returning to pre-disease levels could be a potential biomarker for either treatment efficacy or as a read-out for normal circadian activity in ALL patients following treatment.

### Core and ancillary circadian genes are down-regulated in CML patients upon disease diagnosis

**Figure 3:**
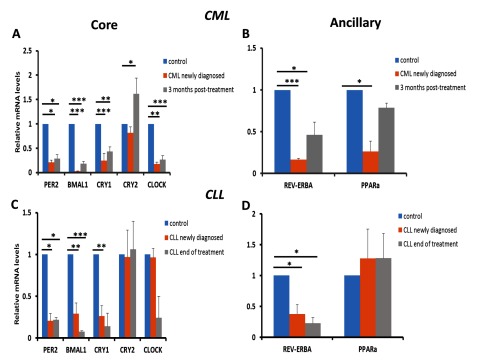
**Core and ancillary circadian genes are down-regulated in PBMCs derived from patients diagnosed with either CML or CLL and show further changes in expression at the end of treatment.** RNA samples from PBMC cells were harvested directly from patients newly diagnosed with AML or ALL, towards the end of treatment for both AML or ALL and during disease relapse for AML patients. The core clock genes including *Per2, BMAL1, Cry1, Cry2 and Clock* and the ancillary clock genes *Rev-ERBa and PPARa* were examined by qRT-PCR and copy numbers were plotted relative to control samples (mean ± SD) following normalization by GAPDH. Statistical differences were determined by student’s t-test assuming unequal variances. Statistical significance at *p < 0.05, **p < 0.01 and ***p < 0.001.

Having analyzed changes in circadian gene expression in both acute forms of leukemia, we next wanted to see how circadian gene expression in chronic forms of leukemia (CML and CLL) contrasted with acute forms of the disease. Initially, we analyzed circadian gene expression in CML patients. For this particular study, 13 CML patients were recruited of which 6 were newly diagnosed with the disease and 7 were patients that had undergone a standard 3-month chemotherapy protocol for CML. Of the core clock genes tested, *Per2* (p < 0.05), BMAL1 (p < 0.001), Cry1 (p < 0.001) and CLOCK (p < 0.001) showed significant down-regulation in expression when compared to expression levels in healthy controls. In addition, the ancillary circadian gene Rev-ERBa (p < 0.001) also showed significant down-regulation in patients newly diagnosed with CML (Figure 3A). The only core circadian gene which did not show significant changes in expression in newly diagnosed patients compared to control samples was Cry2.

### Cry2 is up-regulated above control levels following an initial 3-month course of chemotherapy

Upon the completion of a standard 3-month course of chemotherapy, most of the circadian genes tested showed some increase in expression compared with expression in newly diagnosed patients with the exception being Per2 which did not show any difference (Figure 3A). The interesting observation we saw here was that Cry2 expression in patients that had completed the standard 3-month course of chemotherapy was above levels seen in samples derived from healthy individuals (p < 0.05). This observation could mean one of two things. Either, Cry2 expression was affected by the chemotherapy or Cry2 up-regulation could be a potential marker for treatment efficacy and may be a read-out for the circadian clock returning to normal after treatment.

### The majority of circadian clock genes are down-regulated in CLL patients except Cry2 and PPARa expression which are not affected by disease diagnosis or treatment

In contrast to differential circadian gene expression which was observed in CML patients upon diagnosis and after a first course of treatment, the same was not observed in CLL patients. Only a few of the core clock genes showed statistically significant changes in expression upon disease diagnosis: Per2 (p < 0.05), BMAL1 (p < 0.05) and Cry2 (p < 0.05). For the ancillary clock genes tested, only REV-ERBa showed statistically significant differences upon disease diagnosis (p < 0.01) and upon completion of treatment (p < 0.05). These observations do partially concur with what has been previously reported in CLL patients for Per2 and BMAL1 [21] with the exception being the CLOCK gene. However, our observations for Cry2 in CLL patient samples have not been previously reported.

### Evidence of circadian gene oscillation is present in AML, ALL and CLL patients but is absent in CML patients following cell synchronization

**Figure 4:**
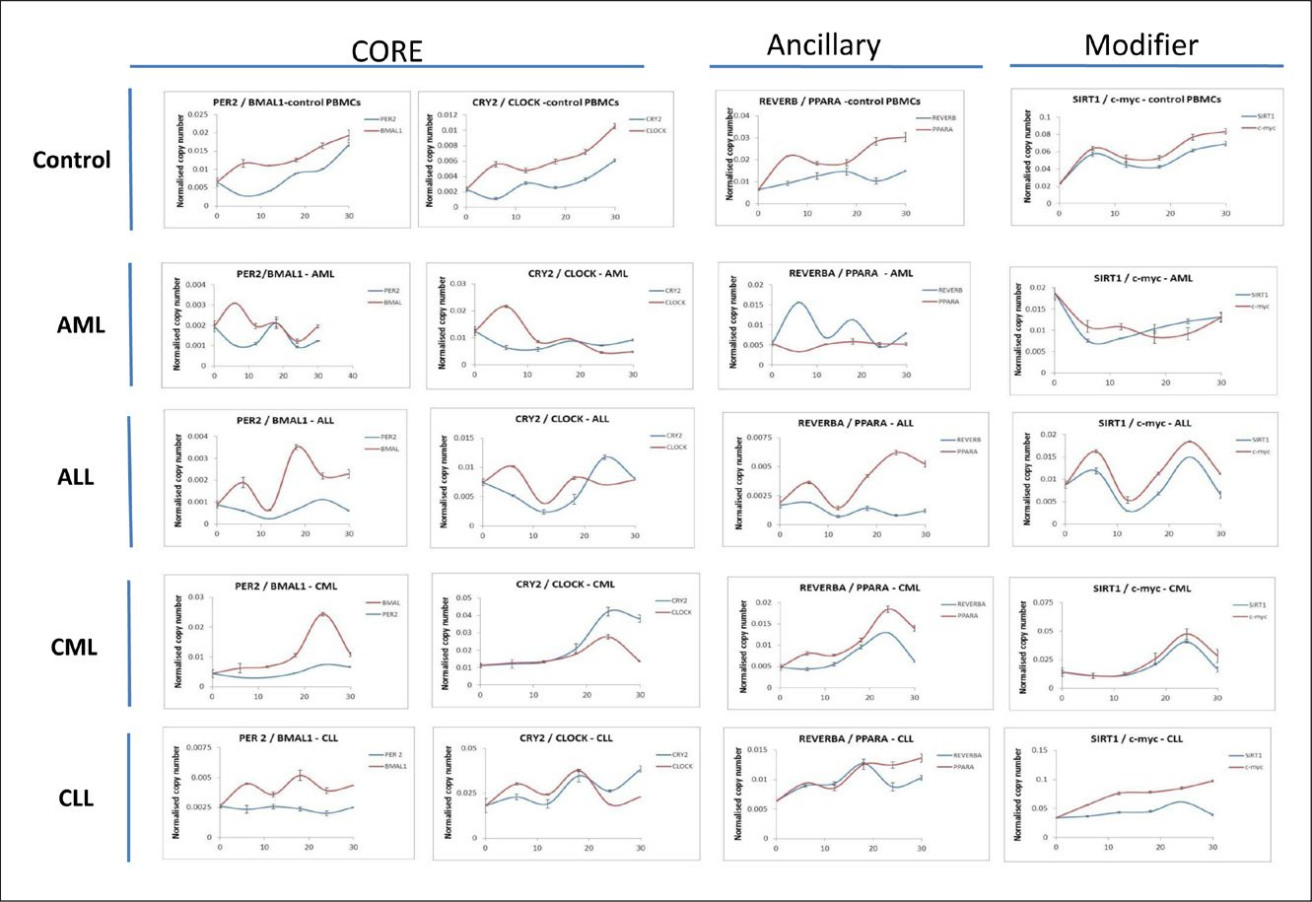
**Circadian oscillations are lost in PBMCs derived from patients diagnosed with CML.** PBMCs derived from newly diagnosed AML, ALL, CML and CLL patients were synchronized using the temperature change method. Following this RNA samples were harvested from the cells every 6hrs over a period of 30hrs and circadian gene expression analyzed by qRT-PCR. Analysis was performed for the following category of genes; Core clock (*Per2/BMAL1, Cry2/clock*), ancillary clock (*Rev-ERBa/PPARa*) and modifier (*SIRT1/c-myc*). PBMCs from each patient category (n = 5) were pooled together before synchronization. Raw Ct values were converted to arbitrary copy numbers and normalized to copy numbers for GAPDH.

Although circadian genes have previously been shown to oscillate in human PBMC cells [22], no study has previously been reported which has looked at the oscillation of core and ancillary clock genes in both acute and chronic forms of leukemia. To study the oscillation we derived PBMCs from patients diagnosed with one of the four categories of leukemia (n = 5 for each category of leukemia). Samples for each category were then pooled and synchronized using the temperature change method. Samples were collected every 6h after synchronization and clock gene expression determined by qRT-PCR. Circadian gene oscillations were then analyzed under the following categories (Figure 4): Core (Per2 vs. BMAL1, Cry2 vs. Clock), Ancillary (Rev-ERBa vs. PPARa) and modifier (SIRT1) and c-myc). The expression of c-myc was measured given that it is a direct downstream target of the SIRT1 transcription regulated complex. As expected, control PBMC samples demonstrated circadian gene oscillation over the 30h analysis period. In addition, both core and ancillary clock genes, Sirt1 and c-myc showed oscillation in AML and ALL patient samples. In CLL patient samples, oscillation of BMAL1, Cry2 and CLOCK were observed (Figure 4). However, oscillation was absent in CML patient samples and this absence was present across all the core and ancillary circadian genes tested as well as the modifier genes Sirt1 and c-myc. Our results suggest that in AML, ALL and CLL patients, evidence of a functional clock are present and that in the case of CML patients, the lack of circadian gene oscillations is representative of a dysfunctional circadian clock.

### Inhibition of Sirt1 using the selective inhibitor EX527 results in some recovery in BMAL1 oscillation in CML patient samples following cell synchronization.

**Figure 5:**
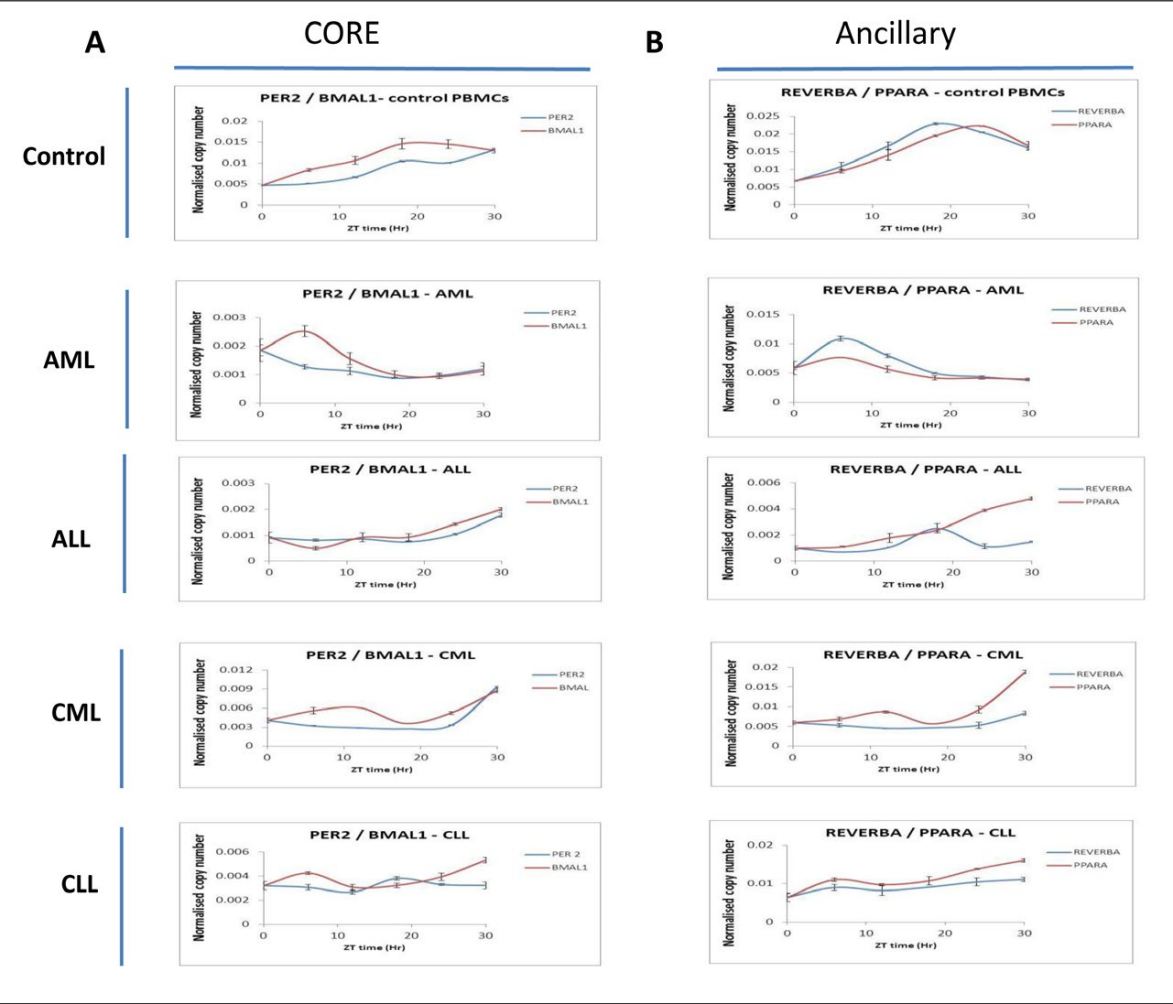
**Treatment with the Sirt1 inhibitor EX527 rescues the oscillation of BMAL1 in CML patients and regulates oscillation of core circadian genes in CLL patients.** PBMCs derived from newly diagnosed AML, ALL, CML and CLL patients were first synchronized and then treated with the SIRT1 inhibitor EX527 (30uM). RNA samples were then harvested every 6hrs after treatment over a period of 30hrs and gene expression analyzed by qRT-PCR for core clock genes **(A)** (BMAL1/Per2) and **(B)** ancillary clock genes (Rev-ERBa/PPARa). PBMCs from each patient category (n = 5) were pooled together before synchronization.

**Figure 6:**
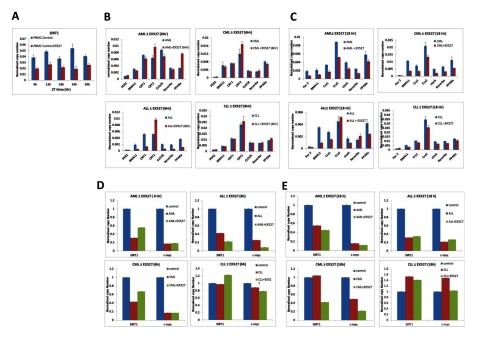
**Treatment with the Sirt1 inhibitor EX527 leads to altered responses in both the early and late-response circadian clock and modifier genes across the different sub-types of leukemia patients.** PBMCs derived from newly diagnosed AML, ALL, CML and CLL patients were first synchronized and then treated with the SIRT1 inhibitor EX527 (30uM). RNA samples were then harvested every 6hrs after treatment over a period of 30hrs and gene expression analyzed by RT-qPCR for core clock genes (BMAL1/Per2) and ancillary clock genes (Rev-Erba/PPARa). PBMCs from each patient category (n = 5) were pooled together before synchronization. **(A)** Sirt1 inhibition by EX527 in control samples over the 30h test period. **(B)** Circadian clock gene expression in the first 6h of treatment with EX527 (early response), **(C)** Circadian clock gene expression after 18h of treatment with EX527 (late-response), **(D)** Expression of clock modifier genes Sirt1 and c-myc after 6h of treatment with EX527 and **(E)** Expression of clock modifier genes Sirt1 and c-myc after 18h treatment with EX527.

Sirt1 is known to regulate genes associated with the circadian clock. As well as being expressed in a circadian manner, a relationship between Sirt1 expression and the acetylation status of BMAL1 and Histone H3 has previously been reported [23]. Separately, Sirt1 has also been shown to bind to CLOCK to promote the de-acetylation of BMAL1 at the CLOCK: BMAL1 chromatin complex [23] (Nakahata Y et al., 2008). We wanted to see whether interfering with Sirt1 activity alters the oscillation of circadian genes in leukemia. Following cell synchronization, samples were treated with the Sirt1-selective inhibitor EX527 at 30 uM, a concentration within the range of concentrations known to selectively inhibit Sirt1 in blood cells [24]. At this concentration, we saw around 50% inhibition of Sirt1 in PBMC samples derived from healthy patients within 6h of treatment as seen by measuring levels of Sirt1 transcript (Figure 6A). In AML patient samples we saw that inhibition of Sirt1 resulted in “fine-tuning” of BMAL1 and Rev-ERBa oscillation. A similar observation was seen in CLL patient samples, where treatment with EX527 resulted in a regularizing of BMAL1 oscillation (Figure 5). The most interesting observation we saw was in CML patient samples, where inhibition of Sirt1 resulted in a return of BMAL1 oscillation which was previously absent in patient samples (Figure 4). Taken together our findings suggest a link between Sirt1 and the oscillation of circadian genes in leukemia.

### In vitro inhibition of Sirt1 significantly modulated circadian clock gene expression in both AML and ALL compared to CML and CLL patients

Having observed that inhibition of Sirt1 played a role in the “fine-tuning” of circadian gene oscillation and in the case of CML led to a complete recovery of BMAL1 oscillation, we next wanted to see whether treatment of patient samples with EX527 altered the relative expression of the circadian genes and how soon after treatment were these changes noticeable. We chose to analyze both 6h and 18h after treatment. Analysis at 6h after treatment would allow us to identify changes in the early-response circadian genes and analysis at 18h after treatment would allow us to identify changes in late-response circadian genes. In AML patient samples we saw an up-regulation of Cry2 and PPARa and a down-regulation of CLOCK (Figure 6B) at 6h following treatment with EX527. In ALL patient samples, similar to what was observed in AML samples, Cry2 was also up-regulated. However, in ALL patient samples all of the other circadian genes tested were all down-regulated within 6h of treatment with EX527 when compared to non-treated patient samples (Figure 6B). In comparison, the response of the circadian genes to 6h of treatment with EX527 in CML and CLL patient samples was virtually identical (Figure 6B) with the exception of Cry2 which was slightly up-regulated in CML. In contrast all of the circadian genes in all patient categories showed down-regulation (apart from Cry2 in ALL patient samples) at 18h following treatment with EX527 when compared to non-treated samples. We also looked at the relative expression of both Sirt1 and c-myc following treatment of patient samples with the inhibitor EX527. In AML, CML and CLL patient samples at 6h following treatment we saw an up-regulation of sirt1 and c-myc expression compared to non-treated samples (Figure 6D). By 18h post-treatment, sirt1 and c-myc expression was inhibited in AML and ALL samples (Figure 6E). Taking together, our data suggests that inhibition of Sirt1 is a feasible approach to modulating circadian gene activity in leukemic patients as part of a chronotherapy-based approach to treatment of acute forms of leukemia.

## Discussion

In this study we have shown that circadian gene expression is affected in different ways depending on the sub-type of leukemia that has been diagnosed and that expression of some of these genes could be used as potential biomarkers to assess therapeutic efficacy. Further we also showed that inhibition of Sirt1 using EX527 may have a role in normalizing BMAL1 oscillation in CML patients. Therefore our work supports the idea of circadian gene expression being altered in different ways depending on the type of leukemia that has been diagnosed and further strengthens the link between leukemia and a disrupted circadian clock.

Although circadian gene expression is known to be disregulated in leukemia, no study has thus far looked at how circadian gene expression changes over the course of leukemia, after treatment and in the case of AML during disease relapse. A recent study has shown that disruption in components of the canonical circadian pathway leads to anti-leukemic effects by depleting leukemia stem cells (LSCs) and impairing proliferation and myeloid differentiation [25]. Here genetic ablation of BMAL1 in mice resulted in impairment in AML maintenance while sparing normal hematopoiesis. In our study, we saw a down-regulation in BMAL1 expression in newly diagnosed AML patients and expression of this gene moved in the direction of control levels in patients who had undergone a relapse of the disease (Figure 2B). Our findings suggests that BMAL1 may play a role during disease by enhancing proliferation and differentiation of LSCs and thus contributing to a more aggressive form of disease which is usually the case during relapse in AML. It would be interesting to isolate LSCs from both AML patients and patients who had undergone a relapse of the disease and compare circadian gene expression and correlate this to LSC proliferation and differentiation studies as this would validate the importance of the circadian clock to LSC biology during relapse of AML.

Changes in circadian gene expression, in particular CLOCK have been previously reported in AML, ALL and CML patients when compared to healthy individuals [9,10,11]. While our findings in AML do concur with these findings, we observed significant down-regulation of the CLOCK gene in patients newly diagnosed with either ALL (Figure 2C) or CML (Figure 3A). The authors of these particular studies sampled patients indigenous to the Far East, whereas the patients recruited for our study were of Middle-eastern heritage in particular those indigenous to Saudi Arabia. It is entirely possible that the differences we saw in our study could be due to genotypic differences between the two study populations which may in turn lead to differences in circadian gene expression in leukemia.

Down-regulation of the core circadian genes BMAL1 and Per2 have previously been reported in patients diagnosed with chronic lymphocytic leukemia (CLL) [21]. Further, these changes in circadian gene expression became more apparent in the CLL group when the patients were further sub-divided into groups working regular shifts vs those working irregular shifts suggesting that changes in circadian gene expression were exacerbated in CLL patients that did not have a regular work pattern. In addition dis-regulated expression of clock-controlled genes such as c-myc and cyclin D1 were also observed. Collectively these findings suggest that aberrant expression of clock genes together with apoptosis and proliferation-related genes may be contributing to the development and maintenance of CLL. Although in our study we did also see significant down-regulation of BMAL1 and Per2 in patients newly diagnosed with CLL (Figure 3C), we also saw significant down-regulation in the core clock gene, Cry2 as well as the ancillary clock gene Rev-ERBa (Figure 3C and Figure 3D). Our findings suggest that the dis-regulation of additional core and ancillary clock genes may well be contributing to the etiology of CLL. Interestingly, we also observed that expression of Cry2 was comparable between CLL patients newly diagnosed with the disease and healthy non-leukemic patients suggesting that not all genes making up the core circadian clock are dis-regulated at least in the case of CLL.

Therapeutic application of circadian rhythms has been used in the area of heart disease to determine time-of-day biomarkers. Classic biomarkers that have been previously used in cardiovascular disease relate to both patient state (e.g. lifestyle factors, age diet, exercise, smoking etc.) and readouts of biological processes (e.g. assessment of transcriptional and/or protein levels) [26,27,28]. Although such biomarkers have traditionally been sampled during the day, more relevant biomarkers termed “chronobiomarkers” are novel biomarkers that are sampled over the course of 24 h, thus sampling both during the day and night, and therefore takes into account the circadian rhythms associated with normal physiological and molecular processes. Currently no such chronobiomarkers have been reported for acute and chronic forms of leukemia, which could be used to assess either treatment efficacy or the re-establishment of normal circadian activity in patients following treatment.

An important observation from our study was how the expression of the *Clock* gene returned to similar levels as those seen in healthy patients following treatment for ALL (Figure 2C). Although our finding does support the potential use of *Clock* gene expression as a novel chronobiomarker in ALL, further studies would have to be performed on larger sample sizes as well as to look at the effect if any of chemotherapy on *Clock* gene expression to rule out the possibility of therapy intervention having a direct effect on the transcription of the *Clock* gene.

Initial therapy for CML usually involves patients being administered tyrosine-kinase inhibitors such as imatinib [29,30] or Dasatinib [31]. Failing this, patients are then usually given an allogeneic stem cell transplant [32]. Until now the effect of chemotherapy on circadian gene expression has not been extensively studied. Terazono *et al* 2008 showed that the treatment of NIH3T3 cells with chemotherapy agent 5-FU for 48 h resulted in a significant reduction of mRNA levels of Period1 (Per1) and Period2 (Per2). Moreover, the injection of 5-FU (2 mg/kg/h) to mice attenuated the oscillation in the expressions of Per1 and Per2 in the liver and suprachiasmatic nuclei, the center of the mammalian circadian clock [33]. This paper describes for the first time that the chemotherapy agent could have an influence on clock gene expression and oscillation. Microarray analyses on imatininb responders in CML have shown widespread changes in cell proliferation markers [34]. Although we cannot completely rule-out the direct effect of imatinib on circadian gene expression, our data does suggest the potential use of circadian markers such as Cry2, Rev-ERBa and PPARa for assessing response to therapy.

Disruption in circadian gene oscillations has previously been shown for the clock genes (Per1, Per2, Per3, Cry1, Cry2 and CKIe) in CML patients, and partial recovery of oscillation in these genes was seen in patients that demonstrated both complete cytogenetic response (CCyR) and major molecular response (MMR) following treatment with imatinib [10]. Although we did also observe disrupted oscillations in Per2 and Cry2 in patients newly diagnosed with CML, the lack of oscillation was not just restricted to these circadian genes but also included additional core clock genes such as BMAL1 and CLOCK, as well as ancillary clock genes such as Rev-ERBa and PPARa (Figure 4). Our findings suggest that a complete disruption of both core and ancillary clocks is manifested in CML.

In addition to looking at circadian gene oscillation in CML, we also looked for oscillation in the other forms of leukemia, AML, ALL and CLL. Interestingly, we did observe some evidence of circadian gene oscillation in AML and ALL patients suggesting that at least in the case of acute leukemia, a functionally intact circadian clock is present. In CLL patients, disruption in oscillation was restricted to Per2, Rev-ERBa and PPARa and oscillation of BMAL1, Cry2 and CLOCK was largely unaffected (Figure 4). Although dysregulation of some circadian genes is known to take place in CLL [21], our findings suggest that the oscillation of other genes remains largely unaffected by the disease.

Another important finding from this study was the “fine-tuning” of core and ancillary clock gene oscillation that as observed when Sirt1 activity was inhibited using the selective inhibitor EX527. In the presence of the inhibitor, BMAL1 and PPARa oscillations were found to be fine-tuned in AML and CLL (Figure 5) patient samples when compared to non-treated patient samples (Figure 4). A role for Sirt1 in regulating circadian gene expression has previously been shown. For example, Sirt1 associates with CLOCK-BMAL1 heterodimers to promote the deacetylation and degradation of Per2 (Asher G et al., 2008). Sirt1 has also been shown to control the circadian expression of genes involved in metabolic pathways in the liver [35]. Although a role for Sirt1 in regulating the circadian clock in leukemia has yet to be established, a role for Sirt1 in cancer has previously been demonstrated. Sirt1 has been shown to regulate cell proliferation and apoptosis through epigenetic modification of tumour suppressor genes such as p53 [36] or oncogenes such as β-catenin [37]. The restoration of BMAL1 oscillation in CML patients following Sirt1 inhibition suggests Sirt1 may be a feasible drug target to restore core clock oscillation in CML patients as part of a chronotherapy strategy for treating CML.

In conclusion, circadian clock gene expression is altered in patients presenting both acute and chronic forms of myeloid and lymphoid leukemia when compared to healthy individuals. Interestingly there is difference in the level of clock gene changes between different patient categories, AML, ALL, CML and CLL. This could be due to the nature of the pathways involved in the malignancy that affect the expression of each clock gene separately in different ways. Further, inhibition of Sirt1 causes the “fine-tuning” of circadian gene oscillation for some types of leukemia and in CML patient’s resulting in the complete recovery of BMAL1 oscillation. Taken together, these data support the notion that SIRT1 has a role in regulating the circadian clock in leukemia and that the targeting of SIRT1 and therefore the molecular clock using chronopharmacological agents such as EX527 could prove to be a novel and effective therapeutic strategy for improving dysfunctional circadian rhythms in leukemia and thus for both improving the effectiveness of existing chemotherapeutic drugs and reducing drug-associated toxicity.

## Dedication

This paper is dedicated to the soul of Dr. Sabhi Rahman (SR) who passed away just after completing the experimental work and writing the paper.

## Additional Files

The additional files for this article can be found as follows:

10.5334/jcr.147.s1 Table 1 Summary of patients details.

**Table 1** Summary of patients details. DOI: https://doi.org/10.5334/jcr.147.s1

10.5334/jcr.147.s2 Figure S1 Expression of Sirt1 and cMyc.

**Figure S1** Expression of Sirt1 and cMyc. DOI: https://doi.org/10.5334/jcr.147.s2
