# Supplementary material for: Differential Expression of Circadian Genes in Leukemia and a Possible Role for Sirt1 in Restoring the Circadian Clock in Chronic Myeloid Leukemia
Source: J Circadian Rhythms. 2017 Apr 28;15:3. doi: 10.5334/jcr.147 (PMC5624060; doi:10.5334/jcr.147)
Supplement: Table 1 — Summary of patients details. [file jcr-15-147-s1.pdf]

Supplementary Table 1

A

No.	Age	Gender	Diagnose	[illegible]
1	33	M	AML	[illegible]
2	59	M	AML	[illegible]
3	73	F	AML	[illegible]
4	68	M	AML	[illegible]
5	37	M	AML	[illegible]
6	30	M	AML	[illegible]
7	29	F	AML	[illegible]
8	36	F	AML	[illegible]
9	76	M	AML	[illegible]
10	74	M	AML	[illegible]
11	63	M	AML	[illegible]
12	27	M	AML	[illegible]
13	15	M	AML	[illegible]
14	47	F	AML	[illegible]
15	71	M	AML	[illegible]
16	46	F	AML	[illegible]
17	63	M	AML	[illegible]
18	62	M	AML	[illegible]
19	38	F	AML	[illegible]
20	48	M	AML	[illegible]
21	55	M	AML	[illegible]
22	52	M	AML	[illegible]
23	55	F	AML	[illegible]
24	25	F	AML	[illegible]
25	55	F	AML	Relapsed
26	33	M	AML	Relapsed

B

No.	Age	Gender	Diagnose	Stage
1	58	F	ALL	New
2	21	F	ALL	New
3	57	M	ALL	New
4	22	M	ALL	New
5	42	M	ALL	New
6	38	F	ALL	New
7	16	F	ALL	New
8	36	M	ALL	New
9	20	F	ALL	NEW
10	20	F	ALL	NEW
11	64	M	ALL	New
12	24	M	ALL	END treatment
13	58	F	ALL	END treatment
14	21	F	ALL	END treatment
15	57	M	ALL	END treatment
16	42	M	ALL	END treatment
17	21	M	ALL	END treatment
18	19	M	ALL	END treatment
19	22	M	ALL	END treatment
20	16	M	ALL	END treatment
21	18	F	ALL	END treatment
22	15	M	ALL	END treatment

C

No.	Age	Gender	Diagnose	Stage
1	54	M	CML	New
2	32	M	CML	New
3	34	F	CML	New
4	62	M	CML	New
5	59	F	CML	New
6	51	F	CML	New
7	69	M	CML	0-3 months
8	53	M	CML	0-3 months
9	41	M	CML	0-3 months
10	43	M	CML	0-3 months
11	62	M	CML	0-3 months
12	48	F	CML	0-3 months
13	41	M	CML	0-3 months

D

No.	Age	Gender	Diagnose	Stage
1	65	M	CLL	New
[illegible]	[illegible]	[illegible]	CLL	New
[illegible]	[illegible]	[illegible]	CLL	New
[illegible]	[illegible]	[illegible]	CLL	New
[illegible]	[illegible]	[illegible]	CLL	New
[illegible]	[illegible]	[illegible]	CLL	New
[illegible]	[illegible]	[illegible]	CLL	New
[illegible]	[illegible]	[illegible]	CLL	New
[illegible]	[illegible]	[illegible]	CLL	New
[illegible]	[illegible]	[illegible]	CLL	New
[illegible]	[illegible]	[illegible]	CLL	New
[illegible]	[illegible]	[illegible]	CLL	END treatment
[illegible]	[illegible]	[illegible]	CLL	END treatment
[illegible]	[illegible]	[illegible]	CLL	END treatment

Table 1: **Summary of patients details:** Table summarizes patients details recruited in the study, their ages, type of diagnosis, gender as well as stage of the disease in different patient categories (A) AML, (B) ALL, (C) CML and (D) CLL
