# Supplementary material for: Differential Expression of Circadian Genes in Leukemia and a Possible Role for Sirt1 in Restoring the Circadian Clock in Chronic Myeloid Leukemia
Source: J Circadian Rhythms. 2017 Apr 28;15:3. doi: 10.5334/jcr.147 (PMC5624060; doi:10.5334/jcr.147)
Supplement: Figure S1 — Expression of Sirt1 and cMyc. [file jcr-15-147-s2.pdf]

Figure S1: **Expression of Sirt1 and cMyc**: QPCR data measuring Sirt1 and cMyc mRNA expression in newly diagnosed and at the end of treatment in AML (A and B) and ALL (C and D) patients subsequently.

Supplementary figures

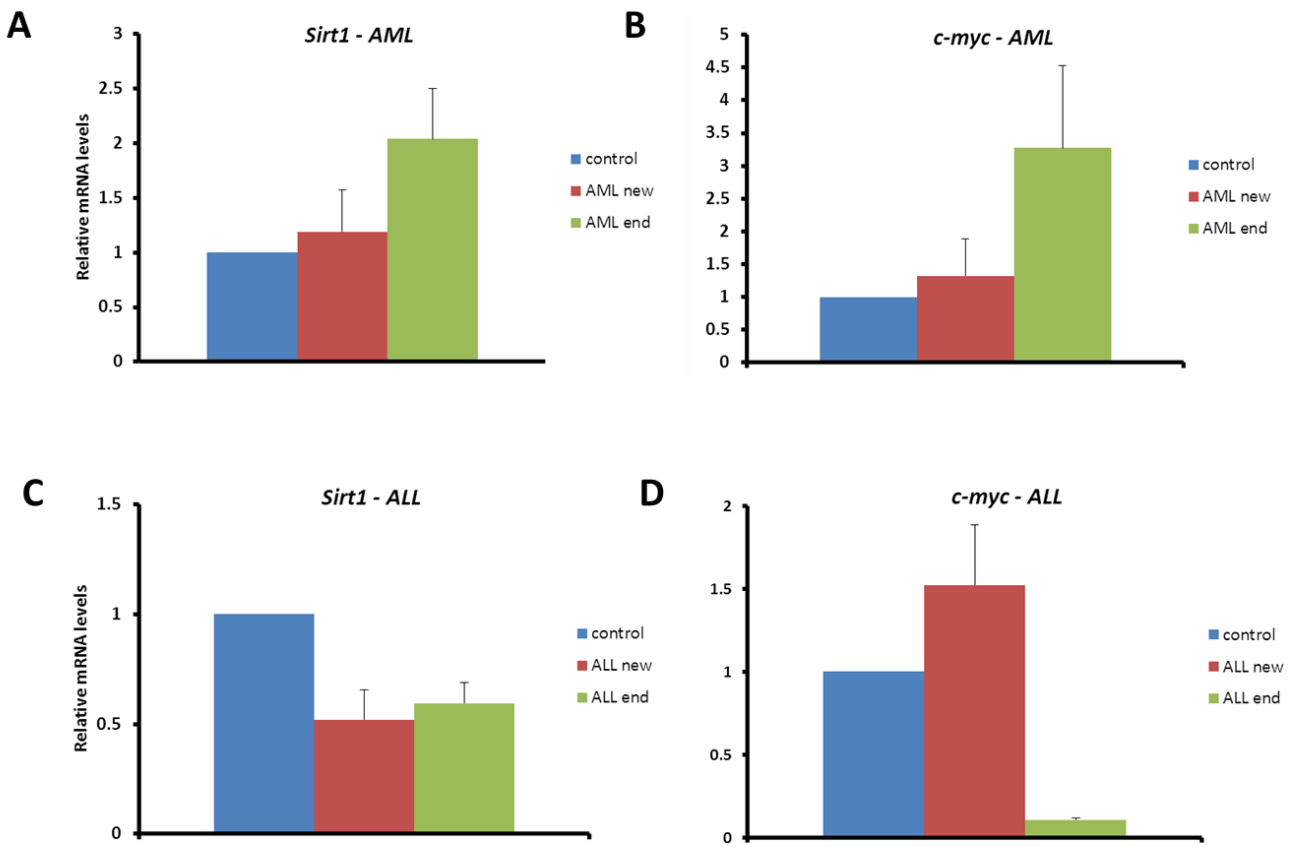

Figure S1
